# Usage of Dermoscopy as an Effective Diagnostic Tool in Pityriasis Alba: A Prospective Observational Study Among Children in a Suburban Hospital in South India

**DOI:** 10.7759/cureus.40271

**Published:** 2023-06-11

**Authors:** Irene N Thomas, Joseph Jenson James, Arishta Bala, Saranya Mohan, Sowmya Dogiparthi, Nithya Priyadharshini Shanmugam

**Affiliations:** 1 Dermatology, Shri Sathya Sai Medical College & Research Institute, Shri Balaji Vidyapeeth, Chennai, IND; 2 Dermatology, Shri Sathya Sai Medical College and Research Institute, Shri Balaji Vidyapeeth, Chennai, IND

**Keywords:** dermoscopy, pityriasis alba, scaling, patches, hypopigmentation

## Abstract

Background: Hypopigmented patches in patients with skin of color are usually a cause of concern. Pityriasis alba is a common skin condition that causes visible patches of hypopigmentation in children and adolescents. In addition to the cosmetic impairment, parents are concerned about the diagnosis of vitiligo and leprosy which also cause hypopigmented patches and have negative social implications. Dermoscopy is a useful diagnostic aid that is acquiring prominence in diagnosing a variety of skin diseases. Few studies exist that validate the use of dermoscopy as an effective tool in the diagnosis of Pityriasis alba.

Objective: To evaluate the effectiveness of dermoscopy by correlating the clinical features of Pityriasis alba with dermoscopic images.

Methods: Hypopigmented patches in 16 patients that were clinically diagnosed as Pityriasis alba were examined with a DermLite DL200 Hybrid dermoscope (Dermlite, CA, USA). All the dermoscopic images were photographically recorded and the findings were noted and correlated with the clinical stages of the disease.

Results: Out of the 40 patches examined in 16 patients, dermoscopic images of white structureless spots, scaling, indistinct borders and normally pigmented hairs were consistently present in all the patches to propose these as the four dermoscopic criteria for the diagnosis of Pityriasis alba. Areas of light brown pigmentation, 17 (42.5%), erythema, 3 (7.5%), and faint pigmented network,11 (27.5%) were the other features noted in some of the patches.

Conclusion: In an ethnic South Indian population where the skin color is predominantly brown, hypopigmented patches are visibly obvious and concerning. Pityriasis alba, Pityriasis versicolor, Vitiligo, Nevus depigmentosus, and Leprosy are the five common conditions seen among children of which Pityriasis alba is the most prevalent. Offering the right diagnosis is essential for the correct management as well as excluding more serious conditions such as leprosy and vitiligo. In this study, Dermoscopy provided a valuable diagnostic aid in achieving this objective.

## Introduction

Understanding the complexity of hypopigmented patches in children is of considerable clinical interest to dermatologists and paediatricians in order to make the right diagnosis and offer proper management. In children in South India where skin colour is predominantly of a darker shade, hypo-pigmented patches in the face are a cause of concern.

In a study conducted among children in South India, the five most prevalent hypopigmentary disorders were found to be Pityriasis alba, Vitiligo, Leprosy, Nevus Depigmentosus and Pityriasis versicolor, with Pityriasis alba having the highest prevalence of 24.7% [1]. Visible hypopigmentation in the face and neck causes parental concern, and dermatologists are frequently consulted to rule out vitiligo and leprosy, two common causes of hypopigmentation in South India that carry the burden of social stigma.

Pityriasis alba is a common, benign skin disorder characterised by round or oval, hypopigmented lesions with fine scales, that is most prevalent in children and adolescent patients with darker complexions [2]. The course of the disease usually lasts for a few months to a year and is mostly asymptomatic. The disorder is usually diagnosed based on a typical history and clinical presentation. Very young children cannot be relied upon to provide an accurate clinical history and clinicians frequently rely on parents to obtain this information. In certain instances, in addition to the patient's history and physical examination, clinicians have to depend on laboratory and diagnostic tests.

Dermoscopy is gaining importance as a reliable non-invasive tool in the diagnosis of several dermatological diseases. In recent years, there has been a surge of interest in the use of dermoscopy for various pigmentary disorders. However, few studies have been published on the use of dermoscopy for the diagnosis of hypopigmentary disorders in children. To the best of our knowledge, no study exists so far on the use of dermoscopy for the diagnosis of Pityriasis alba alone. The aim of this study was to establish the use of dermoscopy as an effective tool for the diagnosis of Pityriasis alba in order to aid clinicians in making the right diagnosis and subsequently to plan the appropriate management.

## Materials and methods

After approval by the Institutional Ethical Committee at Shri Sathya Sai Medical College & Research Institute, Chennai, India, a prospective observational pilot study was conducted among paediatric patients who presented with hypopigmented patches in the face suggestive of a clinical diagnosis of Pityriasis alba.

The inclusion criteria were children up to 12 years of age who had not taken treatment for their disease. After informed consent from the parents, a detailed history with specific attention to Atopic dermatitis, Vitiligo, and Leprosy was taken, and a complete dermatological examination was performed. Those who had taken treatment for the condition were excluded from the study. Sixteen patients who qualified were recruited for the pilot study.

In each patient, lesions were classified according to their number, location, size, shape, scaling, and colour. The Fitzpatrick skin type of each patient was noted. Tests for skin sensation over each patch were elicited. The skin scrapings from the patches were collected, and placed on a slide with potassium hydroxide (10% KOH) and examined under low-power microscopy for fungal hyphae and spores. A diascopic examination was performed with a glass slide over the patches to note whether blanching was present in the surrounding skin to rule out Nevus anemicus. All patches were also examined with a Wood’s lamp in a dark room to differentiate from other hypopigmentary disorders such as Vitiligo and Pityriasis versicolor.

Dermoscopy was performed over all 40 patches examined in the 16 patients with a handheld dermoscope (DermLite DL200 Hybrid; Dermlite, CA, USA) under both polarised and non-polarised light. All the dermoscopic images were captured with an Android phone camera attached to the dermoscope, along with the date and time. The photographic clinical image of the patch with the corresponding dermoscopic image was appropriately filed. In addition, the dermoscopic findings were entered manually into the patient's proforma. Particular attention was paid to noting the degree of hypopigmentation, hair colour, scaling, and borders. Patients were given appropriate treatment and follow-up advice for three monthly visits.

## Results

Table 1 presents the demographic data of the patients studied. The total number of patients recruited for this pilot study was 16. They were in the age group in the range of three to 12 years with a mean age of 8.65. A slight female preponderance was seen with nine females and seven males.

**Table 1:** Demographic details

Gender	Frequency	Percentage	Mean Age
Male	7	43.75%	-
Female	9	56.25%	-
Total	16	-	8.6

Table 2 provides the data related to history. Six children had a history of Atopic dermatitis and one had a family history of Vitiligo. Most of the children were of the darker skin type of Fitzpatrick Type 4 to 5 which is the predominant skin colour of the ethnic population in South India. In the 16 patients recruited for the study, most of the children had more than one patch. The number of patches in each participant ranged from 1 to 4 totalling 40 patches.

**Table 2:** History, associated conditions, and Fitzpatrick skin types

S.no	Associated Conditions	Fitzpatrick skin type iv	Fitzpatrick skin type v
1	Atopic dermatitis	3 (18.75%)	3 (18.75%)
2	Vitiligo	1 (6.25%)	
3	Hansens Disease	Nil	Nil
4	Miscellaneous	5 (31.25%)	4 (25%)
Total(n=16)		9 (56.25%)	7 (43.75%)

Table 3 displays the location of patches within the face.

**Table 3:** Anatomical distribution of facial patches

S.no	Location of patch	Frequency
1	Cheek	25 (62.5%)
2	Forehead	1 (2.5%)
3	Malar	10 (25%)
4	Mandibular	4 (10%)

Table 4 displays the clinical characteristics of the patches that include borders, surface, scaling, and colour.

**Table 4:** Clinical characteristics of patches

S.no	Clinical characteristics	Nature	Frequency
1	Borders	Indistinct border	40 (100%)
		Well defined border	Nil
2	Surface	Even surface	35 (87.5%)
		Papular surface	5 (12.5%)
3	Scaling	Present	37 (92.5%)
		Absent	3 (7.5%)
4	Colour	Hypopigmented	35 (87.5%)
		Erythematous	5 (12.5%)

According to the clinical presentation, the patients were categorized into three stages, the early erythematous papular stage, the hypopigmented scaly stage, and the resolving non-scaly stage. The dermoscopic images of the patches were noted in detail for each patch. Table 5 shows the dermoscopic features of the patches correlated with their clinical staging. Descriptive statistics for numeric variables were represented as mean and for categorical variables as numbers and percentage values.

**Table 5:** Correlation of clinical stages with dermoscopy

	Erythematous Papular stage (n=2) Patches (5)	Hypopigmented scaly stage (n=12) Patches(32)	Residual non scaly stage (n=2) Patches(3)	Total
Superficial white scales	5	32	3	40 (100%)
Hypopigmented structureless areas	5	32	3	40 (100%)
Indistinct borders	5	32	3	40 (100%)
Hair within the patch (Normal colour)	5	32	3	40 (100%)
Areas of light brown pigmentation	2	13	2	17 (42.5%)
Erythema	3	0	0	3 (7.5%)
Faint pigmented network	2	9	0	11 (27.5%)

## Discussion

Pityriasis alba is a common dermatosis that commonly affects children and adolescents. It is characterised by a multistage course and spontaneous remissions and relapses whose aetiology is unknown [3]. The prevalence of Pityriasis alba in South India has been reported to be 24.7% while in other tropical countries such as Brazil and Nepal, the prevalence of pityriasis alba was only 9.9 % and 5.2% [1,4,5].

The age of the patients in the study was between three to 12 years with a mean age of 8.6% with seven males (43.75%) and nine females (56.25%). Fitzpatrick skin type 4 was seen in nine patients (56.25%) and Type 5 in seven patients (43.75%). Particular attention was given to history to note the association with Atopic dermatitis, Leprosy, and Vitiligo. Six children (37.5%) had a history suggestive of Atopic dermatitis, one child had a family history of Vitiligo (6.25%), whereas no past history or family history of Leprosy could be elicited in any of the study participants.

Prior studies regarding the clinical features revealed certain similarities and differences with our study. Vinod et al. have noted that Pityriasis alba lesions commonly occur on the face in 91% of their cases [6]. This was similar to the findings in our patients where all the lesions were in the face, namely, cheek 25 (62.5%), forehead (12.5.%), malar 10 (25%), and mandibular 4 (10%) areas. Pityriasis alba typically manifests with multiple patches rather than singly. Thus, in our study population of 16 patients, we were able to identify 40 patches (Table 2). The characteristic clinical features of scaling were found in 40 out of 40 patches (100%), whereas all patches exhibited a lack of distinct borders. Despite the fact that hypopigmentation was observed in all regions, there were variations in the colour tone. Thirty-five lesions displayed a paler tone compared to the surrounding brownish skin colour of Fitzpatrick Type 4 to 5, whereas five patches were erythematous, which could be due to the disease being observed at an early stage (Table 4).

Since a 10% KOH smear did not reveal any fungal elements or spores in any of the patches, Pityriasis versicolor was eliminated as a differential diagnosis for hypopigmented patches in children. On examination with Wood's lamp, none of the spots fluoresced or became accentuated excluding Vitiligo. Tests of skin sensation are essential for ruling out leprosy in hypopigmented areas. In our study, all our patients (mean age 8.9) were able to appropriately respond when tested for skin sensation in all patches.

**Figure 1:**
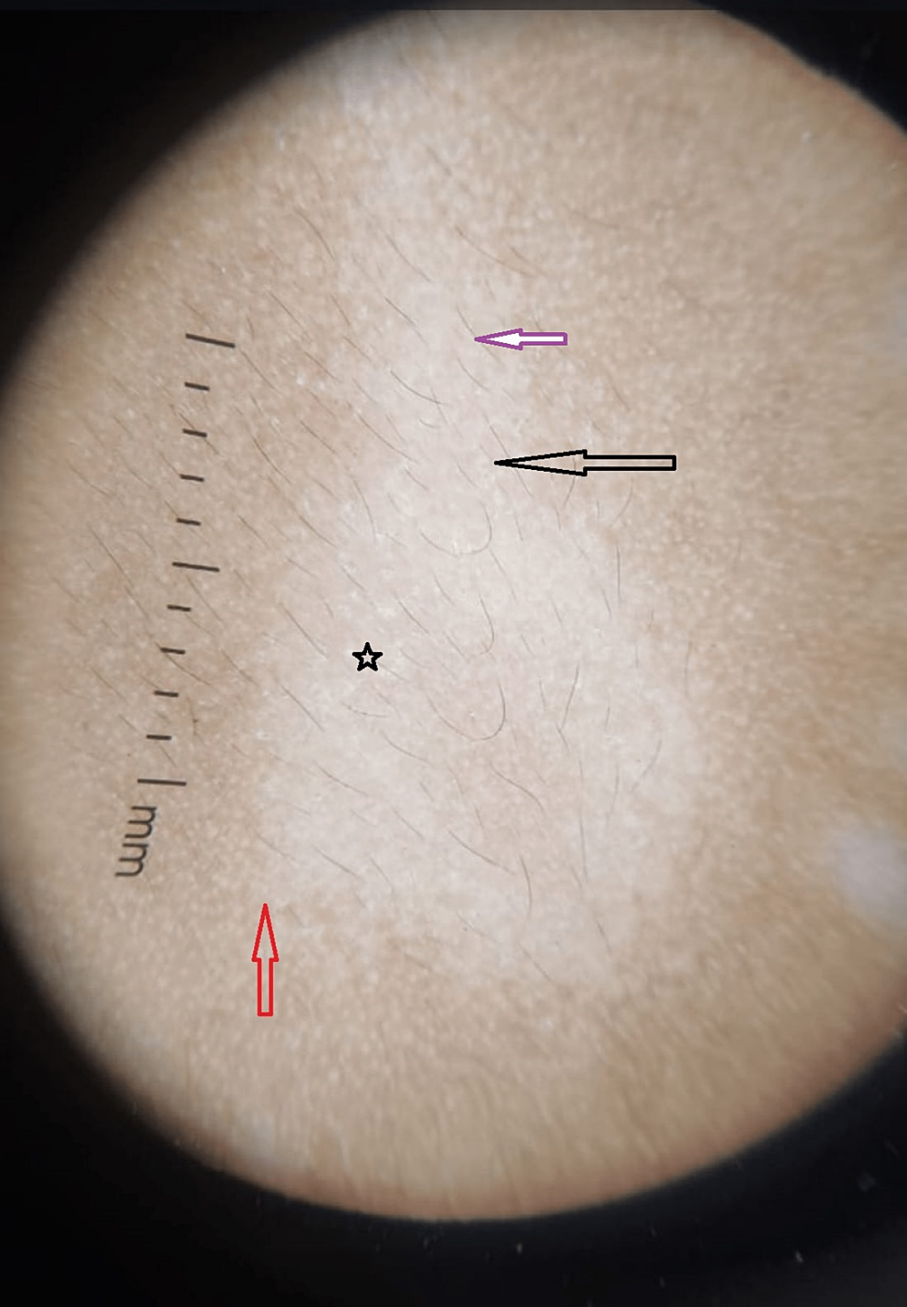
Typical dermoscopic image showing four distinct patterns The image shows a white structureless area (black arrow), scaling (black star), ill-defined borders (red arrow), and pigmented hairs (red arrow).

Typically, Pityriasis alba progresses through a series of stages, starting with an early papular stage, followed by a hypopigmented patch with fine surface scales, and finally resolving as post-inflammatory hypopigmented, non-scaly macules [7]. Four distinct dermoscopy image patterns of scaling, hypopigmented structureless areas, normal hair colour, and indistinct borders were consistently observed in all of the examined patches (Figure 1).

**Figure 2:**
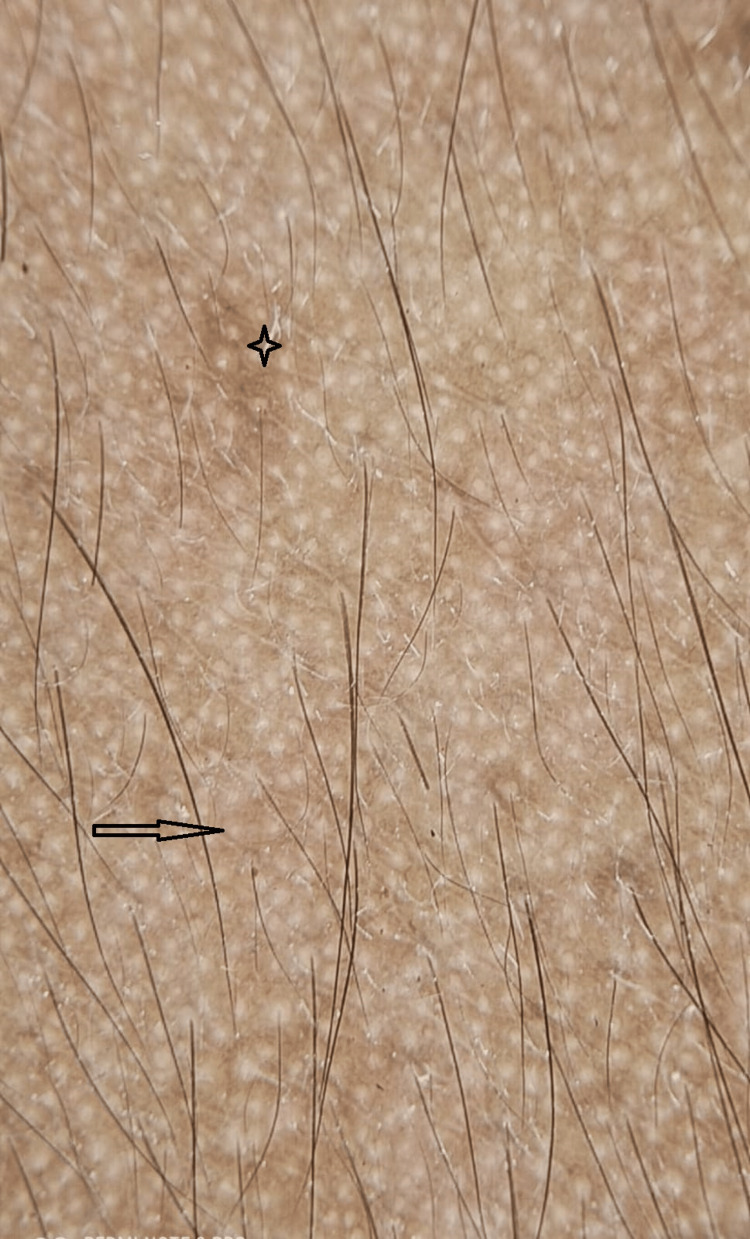
Dermoscopic image showing areas of brown pigmentation The black arrow shows brown pigmentation with a faint pigmented network (black star)

Light brown pigmented areas were observed in 17 patches (42.5%), with a diffuse pigmented network in 11 (27.5%) patches and erythema in three (7.5%) patches. (Figure 2, Table 5).

**Figure 3:**
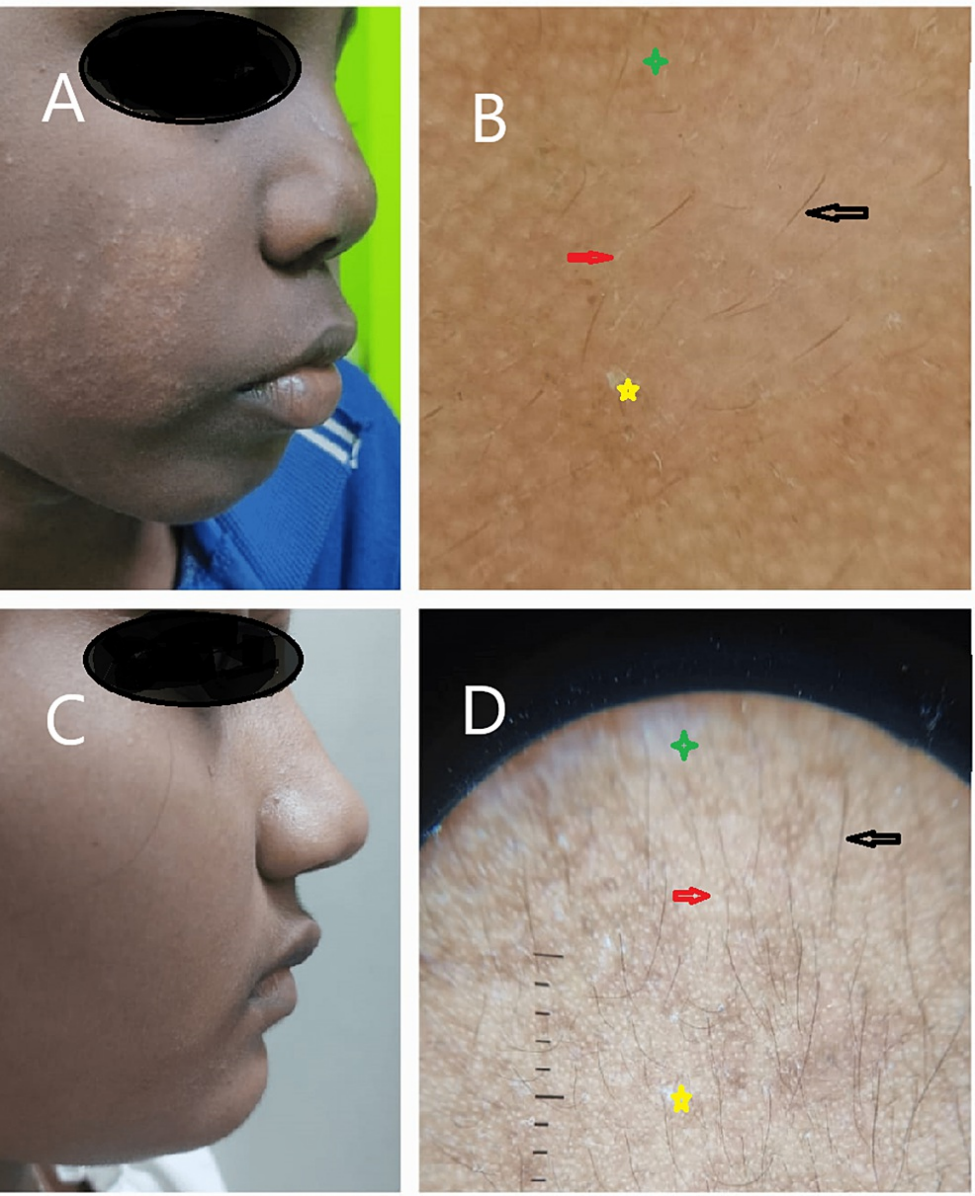
Early erythematous stage images A: A 10-year-old child with early erythematous papules on the face. C:  An 8-year-old child showing hypopigmented papules. B&D: Dermoscopy image shows normal pigmented hair (black arrow), scales (yellow star); white structureless areas (red arrow); indistinct borders (green star).

**Figure 4:**
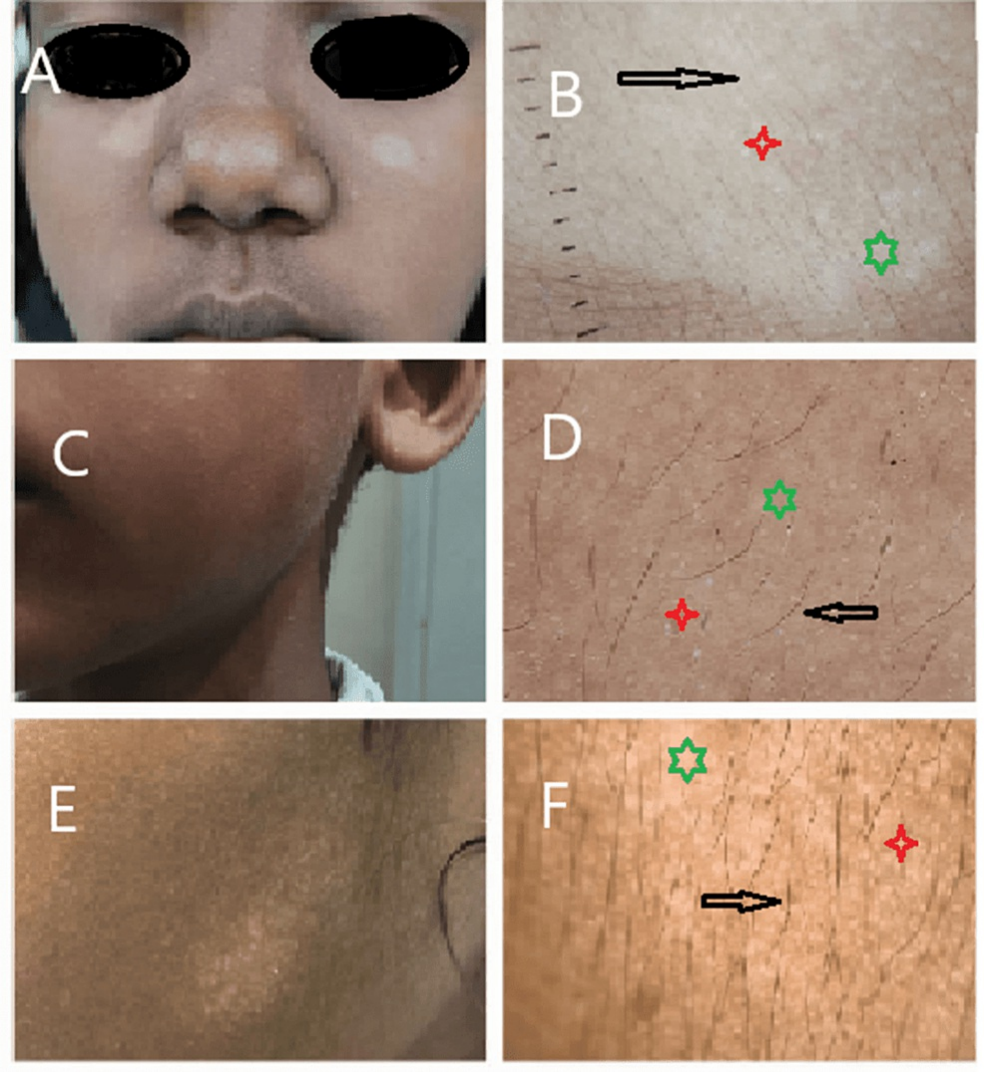
Hypopigmented scaly plaque A: An 8-year-old child with three hypopigmented scaly patches. C: A 6-year-old child with five hypopigmented scaly patches. E: A 6-year-old child with multiple hypopigmented scaly patches. B, D, F: Dermoscopy image shows normal pigmented hair (black arrow); white structureless areas (red star); indistinct borders (green star).

In two of our patients, the early papular lesions were clearly noticed (Figure 3) while 13 patients revealed hypopigmented patches with mild scaling (Figure 4). 

**Figure 5:**
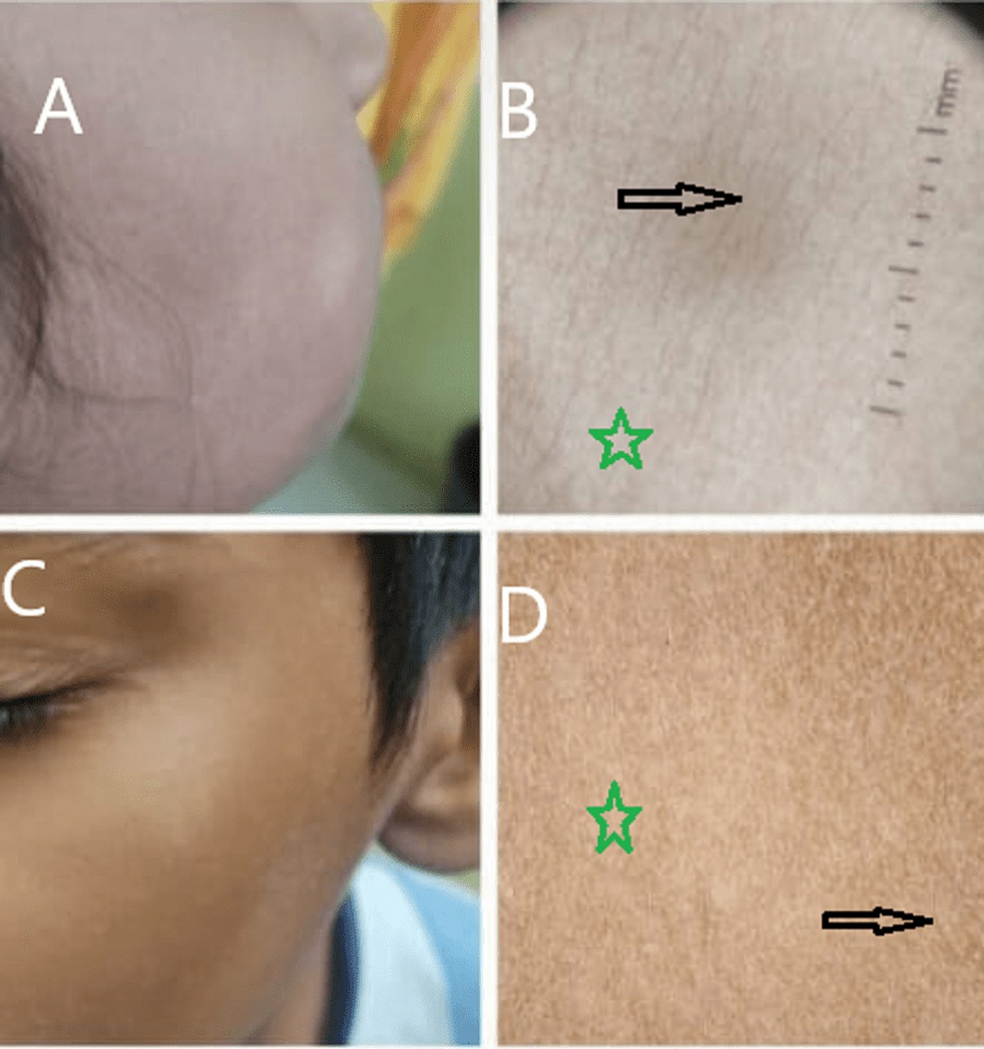
Resolving macular stage images A: A 5-year-old child with resolving patches on the cheek. C: A 10-year-old child with resolving patches in the malar area. B&D: Normal pigmented hair (black arrow); indistinct borders (green star).

Residual post-inflammatory hypopigmented macules without scaling were evident in two of the patients (Figure 5, Table 5).

In a recent study, Vinay and Ankad proposed a dermoscopic diagnostic algorithm for hypopigmented skin disorders in ethnic skin [8]. According to the algorithm, the absence of a white glow rules out vitiligo, and the absence of scaling rules out nevus depigmentosus and leprosy. The pattern of distribution of scales in the patch also helps to distinguish Pityriasis versicolor from Pityriasis alba. The algorithm states that Pityriasis versicolor is characterised by the accumulation of double-edged scales in the skin's creases, whereas in Pityriasis alba, the scaling is uniform and fine and does not accumulate in any particular location. In the patches examined, there was no white glow that eliminated vitiligo and scaling was present that excluded leprosy. The scales were fine and not localised to skin creases, distinguishing Pityriasis alba from Pityriasis versicolor. This dermoscopic algorithmic approach aided us in excluding Vitiligo, Leprosy, and Pityriasis versicolor, the most common differential diagnoses in children with ethnic skin.

In our study, all patients with the clinical diagnosis of Pityriasis alba showed four characteristic features such as scaling, hypopigmented structureless areas, normal hair colour, and indistinct borders in dermoscopy. Based on these findings, we propose these four features as dermoscopic diagnostic criteria of Pityriasis alba (Table 5). Two other studies have also demonstrated these features.

In the study of 16 patients with Pityriasis alba in Jordan, demoscopic features revealed hypopigmented macules with ill-demarcated areas and fine white scales distributed within and outside the macules, with hair inside the patches being of normal colour in all the patches. In addition, 30% demonstrated erythema within and surrounding the patches [9]. In a recent study by Ankad et al, white structureless areas were seen in all of their 30 (100%) patients with Pityriasis alba, with indistinct borders in 86% and fine scales in 70% [10]. They have also reported that 70% of their patients showed fine brown pigmentation while one patient (3.3%) demonstrated a satellite lesion. Our study did not reveal any satellite lesions, though areas of light brown pigmentation were seen in 17 patches (42.5%) and a faint pigment network was noted in 11 patches ( 27.5%). In two patients in the resolving stage, scales were not visible clinically but were present on dermoscopic examination. This was a significant finding in our study.

Although histopathology is the gold standard for diagnosing many skin conditions, Pityriasis alba lacks remarkably distinctive histopathological characteristics. Microscopic features of a mild nonspecific dermatitis with decreased melanin production are the usual finding in Pityriasis alba [11]. In children, an invasive investigation such as a skin biopsy to establish a diagnosis of a benign skin condition such as Pityriasis alba is often unnecessary.

Pityriasis alba is a self-limiting disorder, and with mild topical steroids, most of the lesions resolve over a period of weeks to a few months. On follow-up for three months, most of the patches in our patients had resolved to normal color or as non-scaly macules confirming the diagnosis of Pityriasis alba retrospectively. Regular monthly follow-ups were advised in case patches persisted in order not to miss a diagnosis of indeterminate leprosy.

Limitations

Being a pilot study, the smaller sample size was its limitation. This study needs to be expanded to a larger sample size to enable statistical calculation of sensitivity and specificity. This would further establish the strength of dermoscopy as a valid diagnostic tool in the diagnosis of Pityriasis alba.

## Conclusions

A clinical diagnosis of Pityriasis alba relies predominantly on the history and skin examination. Since the clinical appearance of hypopigmented patches in the differential diagnosis of Pityriasis alba are often closely similar, a more conclusive tool to aid in diagnosis is necessary. In our study, we were able to demonstrate that dermoscopy was a useful clinical diagnostic aid that fulfilled this purpose.

Resolution of the patches also served as a retrospective confirmation of the diagnosis of Pityriasis alba in our patients. Occasionally, non-scaly macules may persist for a prolonged period of time before complete restoration to normal color. In tropical countries where patients with leprosy are still encountered, it is important not to miss the diagnosis of indeterminate leprosy that presents as unresolved hypopigmented patches without complete loss of sensation. Such patients need to be followed regularly. Slit skin smears or a skin biopsy may be performed in order not to miss the diagnosis of indeterminate leprosy and begin early treatment.

In a clinical setting where the skin color is predominantly of a darker complexion, hypopigmented patches concern parents of children because of the visible disfigurement and the apprehension of the diagnosis of vitiligo or leprosy. Our study demonstrates that dermoscopy plays a valuable role as a diagnostic tool to effectively diagnose Pityriasis alba amidst the differential diagnosis of various hypopigmented disorders in ethnic children.
